# Three-phase hierarchical model-based and hybrid inference

**DOI:** 10.1016/j.mex.2023.102321

**Published:** 2023-08-06

**Authors:** Svetlana Saarela, Petri Varvia, Lauri Korhonen, Zhiqiang Yang, Paul L. Patterson, Terje Gobakken, Erik Næsset, Sean P. Healey, Göran Ståhl

**Affiliations:** a Faculty of Environmental Sciences and Natural Resource Management, Norwegian University of Life Sciences, P.O. Box 5003, NO-1432, Ås, Norway; b School of Forest Sciences, University of Eastern Finland, P.O. Box 111, Joensuu FI-80101, Finland; c USDA Forest Service, Rocky Mountain Research Station, 507 25th St, Ogden, UT, USA; d USDA Forest Service, Rocky Mountain Research Station, 240 W Prospect, Fort Collins, CO 80526, USA; e Faculty of Forest Sciences, Swedish University of Agricultural Sciences, SLU Skogsmarksgränd 17, SE-90183, Umeå, Sweden

**Keywords:** Forest resources assessment, Remotely sensed data, Statistical inference, Superpopulation-based inference, Three-phase Hierarchical Model-based and Hybrid Inference

## Abstract

Global commitments to mitigating climate change and halting biodiversity loss require reliable information about Earth's ecosystems. Increasingly, such information is obtained from multiple sources of remotely sensed data combined with data acquired in the field. This new wealth of data poses challenges regarding the combination of different data sources to derive the required information and assess uncertainties. In this article, we show how predictors and their variances can be derived when hierarchically nested models are applied. Previous studies have developed methods for cases involving two modeling steps, such as biomass prediction relying on tree-level allometric models and models linking plot-level field data with remotely sensed data. This study extends the analysis to cases involving three modeling steps to cover new important applications. The additional step might involve an intermediate model, linking field and remotely sensed data available from a small sample, for making predictions that are subsequently used for training a final prediction model based on remotely sensed data:

• In cases where the data in the final step are available wall-to-wall, we denote the approach three-phase hierarchical model-based inference (3pHMB),
• In cases where the data in the final step are available as a probability sample, we denote the approach three-phase hierarchical hybrid inference (3pHHY).

## Graphical abstract

**Figure:**
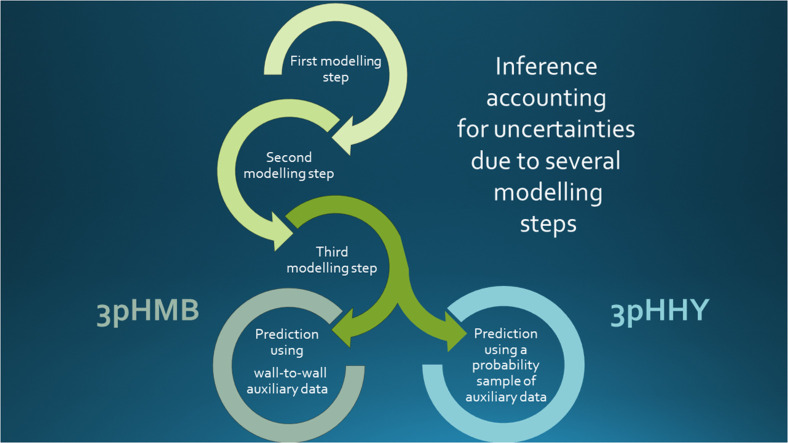

Specifications table

**Table:** 

Subject area:	Environmental Science
More specific subject area:	*Statistical Inference based on Earth Observation data*
Name of your method:	*Three-phase Hierarchical Model-based and Hybrid Inference*
Name and reference of original method:	*N.A.*
Resource availability:	*3pHMB_3pHHY_MC-based_valid.Rmd*

## Method details

### Background

Assessment of state and change of forest biomass has become important worldwide due to the large carbon dioxide fluxes incurred by deforestation and afforestation, as well as by tree growth and harvesting in managed forests (e.g., [1]). Intergovernmental Panel on Climate Change (IPCC) guidance specifies that parties must report changes in biomass carbon stocks as well as uncertainties of the reported figures. Since large parts of the world's forests are inaccessible, applying remotely sensed (RS) data is an important option for such assessments (ibid.). However, the accuracies of the assessments should be quantified using objective and consistent methods (e.g., [2]).

A standard model-based approach to forest biomass assessment is to specify and estimate a model that links biomass measured in the field with RS metrics. The estimated model is then applied across the forest area of interest, whereby biomass predictions and their uncertainties can be obtained utilizing standard methods of model-based inference (e.g., [3]). Uncertainties arise since a model cannot perfectly predict the actual biomass conditions. In some cases, several models are needed to make biomass assessments feasible. For example, models are often applied to predict the biomass of individual trees in the field from measurements of their diameters and heights. Subsequently, these predicted biomasses are used for developing models linking field biomass at the level of sample plots with RS metrics. In this case, the overall uncertainty arises from two modeling steps (e.g., [4]).

Remote sensing methods that utilize laser measurements have revolutionized forest resource assessments [5], [6], [7]. These methods provide information about the 3D structure of forests, which is closely linked to biomass. However, laser measurements are expensive, and thus in many cases they cannot be conducted wall-to-wall but only through sample coverage [8]. Important examples include the strip samples of laser measurements conducted in Alaska [9], and the samples of laser footprints from NASA's Global Ecosystem Dynamics Investigation (GEDI; [1,10] and the Ice, Cloud, and land Elevation Satellites 1 and 2 (ICESat-1 and −2; [11], [12], [13], [14], [15], [16], [17]. For many of these applications, existing methods for assessing uncertainties using frameworks such as model-assisted estimation (e.g., [9]), hybrid inference (e.g., [1,11]), and hierarchical model-based inference (e.g., [10]) have been sufficient.

Sometimes the available data structure requires additional levels in the hierarchy. New methods that utilize several RS-based models in hierarchical structures call for further development of methods to assess uncertainties. An example application involves modeling plot level biomass from field measurements, linking these with laser measurements through a second model, and utilizing laser-based predictions of biomass at sample locations as substitutes for field data in estimating a third model for biomass prediction based on RS data available wall-to-wall, such as data from the Landsat satellite [18]. In this case, three hierarchically nested modeling steps contribute to the overall uncertainty of the biomass prediction for the study area. Ideally, the different datasets involved should stem from about the same time point Hou et al. [19].

Note the difference between this type of hierarchically nested models and the hierarchy of data and models applied in standard multi-level modeling (e.g., [20]). In our case, the target response variable in the first modeling step is replaced by model predictions of the response variable in the subsequent modeling steps. In standard multi-level modeling, the response variable does not change, and multi-level modeling (also called mixed-effects modeling) refers to handling groups of observations, within which the observations are dependent.

This article aims to develop and demonstrate predictors and methods for uncertainty assessment for model-based inference involving three hierarchically nested modeling steps. If data in the final step are available wall-to-wall, we denote the method three-phase hierarchical model-based inference (3pHMB). If data in the last step are available from a probability sample, we denote the method three-phase hierarchical hybrid inference (3pHHY). The methods are similar in the first three steps, and differ only in the last step (see the Graphical Abstract).

### Overview

In the following sections, we first derive and present the formulas needed for applying the 3pHMB and 3pHHY frameworks. Secondly, we provide further theoretical support for the approach through an analysis based on a multivariate superpopulation model; in this section, we also demonstrate important consequences of multi-stage hierarchical modeling. Lastly, we validate our theoretical results through Monte Carlo simulation.

### Details of three-phase hierarchical model-based inference (3pHMB)

We base our description of 3pHMB inference on an example from forest inventory aiming at assessing the biomass for some large study area. We define our population as the grid-cells that tessellate the study area into N units. The size of the grid-cells corresponds to the size of plots utilized in field inventories and to the size of pixels for which RS data can be retrieved. For each grid-cell RS data are available, e.g., multispectral optical satellite data from the Landsat satellite (e.g., [21,22]). With the 3pHMB method, wall-to-wall RS data are used for predicting the biomass in each grid-cell and through averaging across the study area we predict the biomass density. All other data sources, described below, are utilized for providing data for estimating the parameters of this prediction model, which we denote the Landsat model.

When the 3pHMB method is applied, field data are not adequate for immediately estimating the parameters of the Landsat model, but “pseudo-field” biomass data are available from a sample of accurate predictions based on, e.g., airborne laser scanning (ALS) data. This sample dataset is used for estimating the Landsat model parameters. However, to make the ALS-based predictions, the parameters of the ALS model must also be estimated. This model is estimated from a small sample of field biomass assessments with corresponding ALS data.

The biomasses of trees in the field also need to be predicted from models (often called allometric biomass models, e.g., [23]), since direct measurement is expensive and destroys the resource being monitored. Thus, biomass models are typically estimated from small samples of trees, which are cut down and carefully weighed during dedicated research studies (e.g., [24,25]). When applying the 3pHMB method we often aggregate model predictions of tree-level biomass to plot level, e.g., using the method described in Saarela et al. [[4], pp. 11–12, Section “Aggregation of tree-level AGB predictions to plot level”] and in Varvia et al. [[26], Appendix A.1]. In this article, we do not address the details of this aggregation but assume that plot level biomass is either available from aggregation of tree-level predictions (based on field data) or directly predicted from plot level measurements in the field of, e.g., basal area and mean height.

Thus, our study area of interest (AOI) is tessellated into N grid-cells that constitute the population elements. We assume that the objective is to predict the population mean, ${\bar{y}}_{U}$, of grid-cell level aboveground biomass density (AGBD), defined as

$${\bar{y}}_{U}=\frac{1}{N}\sum_{i=1}^{N}y_{i},$$

where U denotes the set of elements in the AOI. The RS data available for this set (e.g., Landsat data) are denoted $P_{U}$, which is a matrix with N observations of t explanatory variables (plus a column of 1′s for a model intercept). The dataset available for estimating the prediction model used in the final stage is denoted $S_{III\;}.\;$It has $n_{III}$ sample elements and contains data of the kind described above (denoted $P_{III}$ for this set) and intermediate RS data (e.g. from ALS) denoted$\;Z_{III}$, which is a matrix with $n_{III}$ observations of qexplanatory variables (plus a column of 1′s). Further, the dataset $S_{II}$ has $n_{II}$ elements and data in the datasets $Z_{II}$ (as above, but with $n_{II}$ observations) and $X_{II}$, which is a matrix with $n_{II}$ observations of h variables (plus a column of 1′s). The latter data are field measurements (such as basal area, and plot averages of tree diameters and tree heights). Finally, the dataset $S_{I}$ has $n_{I}$ observations. It contains field measurements in $X_{I}$ (as above, but with $n_{I}$ observations) and data from actual measurements of AGBD, in the vector $y_{I}$.

For deriving a formula for the variance of the 3pHMB method, we start by defining a univariate linear model F, which links AGBD and field measurements at the grid-cell level, using data from $S_{I}$:

(1) $$F:Y_{i}=x_{i}\beta +\epsilon_{i},\;\epsilon_{i}∼N\left(0,\omega^{2}\right),$$

where $Y_{i}$ is the variable of interest (AGBD), $x_{i}$ is a (h+1)-length row vector of explanatory variables (field measurements) with a unit term for the intercept, β is a (h+1)-length column vector of model parameters to be estimated, and $\epsilon_{i}$ is a variability (a.k.a. error) term which we assume follows a normal distribution with zero mean and constant variance $\omega^{2}$.^1^

The model parameters are estimated using the ordinary least squares (OLS) estimator (e.g., [27]):

$$\hat{\beta }={\left(X_{I}^{T}X_{I}\right)}^{-1}X_{I}^{T}y_{I}$$

The estimated model is then used to predict the target variable (AGBD) for the elements in $S_{II}$:

$${\hat{y}}_{F}=X_{II}\hat{\beta }\;.$$

The index F is used to denote that the AGBD predictions in $S_{II}$ are based on the model F (Eq. (1)). The values ${\hat{y}}_{F}$ are predictions of AGBD based on field data, which are subsequently used for estimating the parameters of the model predicting AGBD from ALS data. The ALS model is thus the next model to be addressed in the model hierarchy.

To support the construction of the ALS model, we specify a multivariate model, G${}^{*}$, which links the (h+1)-multivariate response variable of field measurements $X_{i}$ and a (q+1)-length row vector $z_{i}$ of ALS explanatory variables:

$$G^{*}:X_{i}=z_{i}A^{*}+\upsilon_{i}^{*},\;\upsilon_{i}^{*}∼N\left(0,\Delta^{*}\right),$$

where$\;A^{*}$ is a ((q+1)×(h+1)) - matrix of model parameters to be estimated, $\upsilon_{i}^{*}$ is a (h+1)-length multivariate vector of variability terms, which are assumed to be independent, normally distributed, have zero mean, and their variance-covariance matrix $\Delta^{*}$ is of size ((h+1)×(h+1)).

By multiplying each model component in G${}^{*}$ by β we obtain the univariate model ([28], Appendix A.3)

$$X_{i}\beta =z_{i}A^{*}\beta +\upsilon_{i}^{*}\beta ,\;\upsilon_{i}^{*}\beta ∼N\left(0,\beta^{T}\Delta^{*}\beta \right),$$

Denoting $A^{*}\beta$ as α, $\upsilon_{i}^{*}\beta$ as $\upsilon_{i}$, and $\beta^{T}\Delta^{*}\beta$ as $\delta^{2}$, we obtain the following model, which we denote as G:

(2) $$G:X_{i}\beta =z_{i}\alpha +\upsilon_{i},\;\upsilon_{i}∼N\left(0,\delta^{2}\right)\;.$$

Here, $z_{i}$ a (q+1)-length row vector of ALS explanatory variables with a unit term for the intercept and α is a (q+1)-length column vector of model parameters to be estimated. This model links the expectation of AGBD, due to the model F, with ALS explanatory variables.

The predictions ${\hat{y}}_{F}$ are used as the response variables in the model G to estimate its model parameters, α**,** based on the explanatory variables $Z_{II}$ from $S_{II}$ ([28], Appendix A.3):

$$\hat{\alpha }={\left(Z_{II}^{T}Z_{II}\right)}^{-1}Z_{II}^{T}{\hat{y}}_{F}={\left(Z_{II}^{T}Z_{II}\right)}^{-1}Z_{II}^{T}X_{II}\hat{\beta }=\hat{A^{*}}\hat{\beta \;.}$$

Here, $\hat{A^{*}}={\left(Z_{II}^{T}Z_{II}\right)}^{-1}Z_{II}^{T}X_{II}$ are estimated model parameters from the multivariate model G${}^{*}$.

At this point, we have utilized the actual measurements of AGBD from $S_{I\;}$to predict AGBD for all elements in $S_{II\;}$based on field measurement data. The AGBD predictions for $S_{II\;}$have subsequently been used for training an ALS model (the G model), through which we now can predict AGBD for $S_{III}$:

$${\hat{y}}_{G}=Z_{III}\hat{\alpha }.$$

The predicted values ${\hat{y}}_{G}$ are used for training the model to be applied in the last stage, based on wall-to-wall RS data, i.e., the Landsat model.

In support of constructing the Landsat model, we introduce a second multivariate model, which links the (q+1)-multivariate variable $Z_{i}$ (ALS variables) as a multivariate response variable with a (t+1)-length row vector of Landsat explanatory variables $p_{i}$; the model is denoted Q${}^{*}$:

$$Q^{*}:Z_{i}=p_{i}\Gamma^{*}+e_{i}^{*},\;e_{i}^{*}∼N\left(0,\Theta^{*}\right),$$

where $\Gamma^{*}$ is a ((t+1)×(q+1))-matrix of model parameters, $e_{i}^{*}$ is a (q+1)-multivariate variable of variability terms which are independent, normally distributed, have zero mean and variance-covariance $\Theta^{*}$ of size ((q+1)×(q+1)).

By multiplying model components in Q${}^{*}$ with the model coefficients from G, we obtain the following univariate model

$$Z_{i}\alpha =p_{i}\Gamma^{*}\alpha +e_{i}^{*}\alpha ,\;e_{i}^{*}\alpha ∼N\left(0,\alpha^{T}\Theta^{*}\alpha \right).$$

Denoting $\Gamma^{*}\alpha$ as γ, $e_{i}^{*}\alpha$ as $e_{i}$ and $\alpha^{T}\Theta^{*}\alpha$ as $\theta^{2}$ we obtain the following model, which we denote as Q:

(3) $$Q:Z_{i}\alpha =p_{i}\gamma +e_{i},\;e_{i}∼N\left(0,\theta^{2}\right),$$

where, γ is an (t+1)-length vector of model coefficients to be estimated.

The ALS-based predictions of AGBD, ${\hat{y}}_{G}$, are used for estimating the model parameters, γ**,** using information on explanatory Landsat variables $P_{III}$ from $S_{III}$:

$$\hat{\gamma }={\left(P_{III}^{T}P_{III}\right)}^{-1}P_{III}^{T}{\hat{y}}_{G}={\left(P_{III}^{T}P_{III}\right)}^{-1}P_{III}^{T}Z_{III}\hat{\alpha }={\left(P_{III}^{T}P_{III}\right)}^{-1}P_{III}^{T}Z_{III}\hat{A^{*}}\hat{\beta }=\hat{\Gamma^{*}}\hat{A^{*}}\hat{\beta },$$

where $\hat{\Gamma^{*}}={\left(P_{III}^{T}P_{III}\right)}^{-1}P_{III}^{T}Z_{III}$ are estimated model parameters from the model Q${}^{*}$.

Finally, the estimated Q model is used to predict the AGBD across the entire AOI, i.e., for all the elements in U:

$${\hat{y}}_{U}=P_{U}\hat{\gamma }.$$

The 3pHMB population mean predictor is then:

(4) $${\hat{\bar{y}}}_{U_{3pHMB}}=\frac{1}{N}\sum_{i=1}^{N}p_{i}\hat{\gamma }=\;{\bar{p}}_{U}\hat{\gamma },$$

where ${\bar{p}}_{U}$ is the (t+1)-length row vector of Landsat explanatory variable averages over the AOI.

Table 1 summarizes the description of the datasets and the estimation steps. In the table, the datasets used in the analyses are listed with descriptions of available variables and whether they are used for model training or model application.

**Table 1:** A summary of datasets and estimation steps involved in 3pHMB prediction.

Dataset	Available information	Model training steps	Model application step
The field dataset $S_{I}$	AGBD and field measurements	A model linking AGBD and field measurements (model F)	–
The ALS dataset $S_{II}$	Field measurements and ALS variables	An ALS model, used to predict AGBD from ALS variables (model G)	The model F is applied on the $S_{II}\;$dataset
The Landsat dataset $S_{III}$	ALS and Landsat variables	A Landsat model, used to predict AGBD from Landsat variables (model Q)	The model G is applied on the $S_{III}$ dataset
The AOI, U	Landsat data	–	The model Q is applied to the entire population

**Fig. 1:**
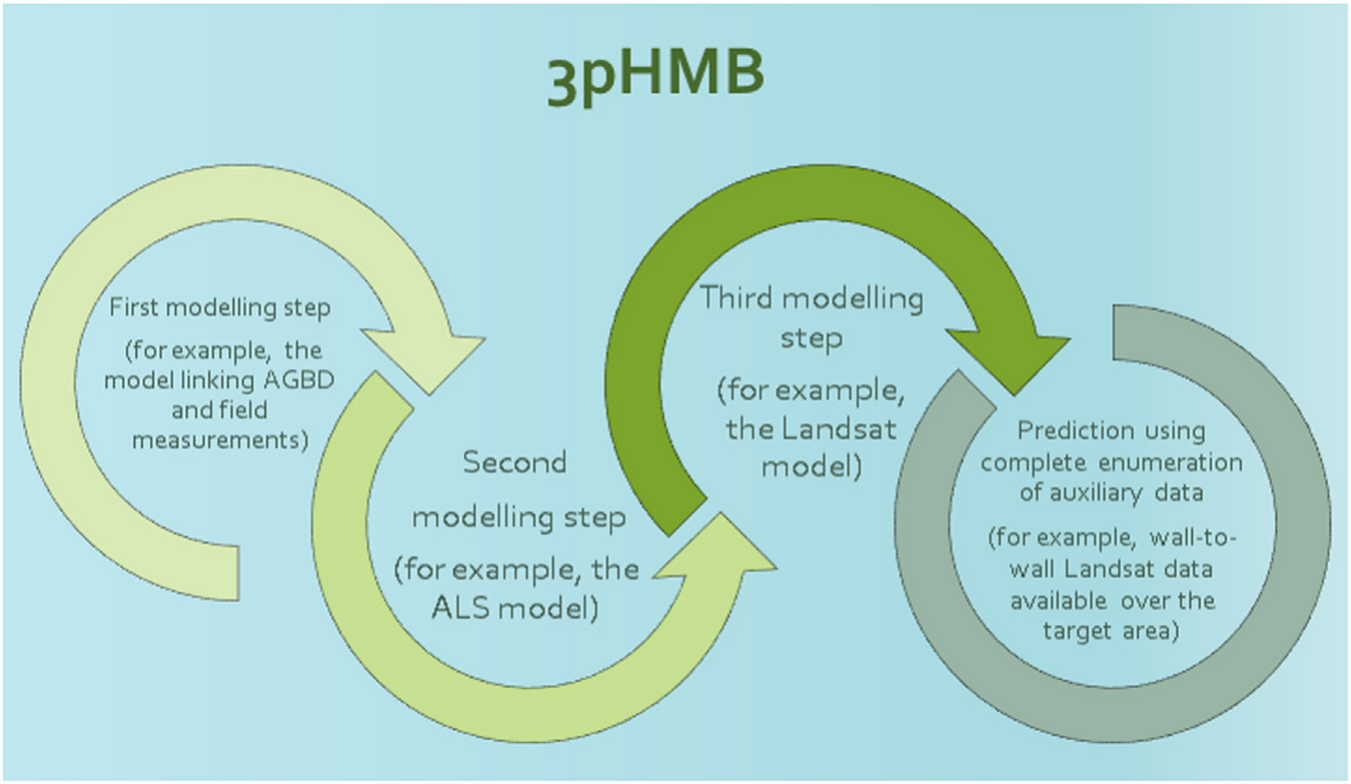
A graphical overview of 3pHMB prediction based on wall-to-wall auxiliary (remotely sensed) data.

Fig. 1 gives a graphical overview of 3pHMB prediction.

### The 3pHMB predictor variance and its estimator

Application of 3pHMB for making predictions, only, across some AOI is fairly straightforward, and does not require all the model formalism from the previous section. What is required is a procedure where the AGBD is first measured on the elements in $S_{I\;}$, then predicted in a stepwise manner for the elements in $S_{II\;},\;S_{III\;}$, and U. However, the formalism introduced in the previous section is needed for assessing the uncertainty associated with predicting the AGBD for the AOI. In this section, we build on the previous section and derive a formula for the variance of the predictor of AGBD, and its corresponding estimator.

For deriving the 3pHMB predictor variance, we begin with its expectation:

$$E\left[{\hat{\bar{y}}}_{U_{3pHMB}}\right]=E\left[{\bar{p}}_{U}\hat{\gamma }\right]={\bar{p}}_{U}E\left[\hat{\gamma }\right].$$

Since independent datasets (normally) are used for the three modeling steps, the estimated parameters in each step are independent and thus

$$E\left[\hat{\gamma }\right]=E\left[\hat{\Gamma^{*}}\hat{A^{*}}\hat{\beta }\right]=\Gamma^{*}A^{*}\beta =\gamma .$$

Thus, the expectation of the predictor of the population mean is

$$E\left[{\hat{\bar{y}}}_{U_{3pHMB}}\right]={\bar{p}}_{U}\gamma .$$

Following the definition, the variance of ${\hat{\bar{y}}}_{U_{3pHMB}}$ is

(5) $$\begin{array}{ccc} V\left({\hat{\bar{y}}}_{U3pHMB}\right) & = & E\left[{\left({\hat{\bar{y}}}_{U3pHMB}-E\left[{\hat{\bar{y}}}_{U3pHMB}\right]\right)}^{2}\right]\\ & = & E\left[{\left({\bar{p}}_{U}\hat{\gamma }-E\left[{\bar{p}}_{U}\hat{\gamma }\right]\right)}^{2}\right]\\ & = & {\bar{p}}_{U}E\left[{\left(\hat{\gamma }-E\left[\hat{\gamma }\right]\right)}^{2}\right]{\bar{p}}_{U}^{T}\\ & = & \bar{p}Cov\left(\hat{\gamma }\right){\bar{p}}_{U}^{T}. \end{array}$$

It can be noted that the covariance of the estimated model parameters is at the core of this expression. Since several modeling steps have been involved in the estimation of the model parameters $\hat{\gamma }$*,* the covariance can be decomposed into the following terms using the law of total covariance [29]:

(6) $$\begin{array}{ccc} Cov\left(\hat{\gamma }\right) & = & Cov\left(\hat{\Gamma *}\hat{\alpha }\right)\\ & = & E_{G}\left[Cov_{Q*}\left(\hat{\Gamma *}\hat{\alpha }\right)\right]+Cov_{G}\left(E_{Q*}\left[\hat{\Gamma *}\hat{\alpha }\right]\right)\\ & = & E_{G}\left[{\hat{\alpha }}^{T}Cov_{Q*}\left(\hat{\Gamma *}\right)\hat{\alpha }\right]+\Gamma *Cov_{G}\left(\hat{\alpha }\right)\Gamma {*}^{T}\\ & = & \alpha^{T}Cov_{Q*}\left(\hat{\Gamma *}\right)\alpha +\Gamma *Cov_{G}\left(\hat{\alpha }\right)\Gamma {*}^{T}+Tr\left[Cov_{Q*}\left(\hat{\Gamma *}\right)Cov_{G}\left(\hat{\alpha }\right)\right], \end{array}$$

where the step from the second last to the last line is obtained from

$$\begin{array}{ccc} E_{G}\left[{\hat{\alpha }}^{T}Cov_{Q*}\left(\hat{\Gamma *}\right)\hat{\alpha }\right] & = & E_{G}\left[\sum_{i=1}^{\left(q+1\right)}\sum_{j=1}^{\left(q+1\right)}{\hat{\alpha }}_{i}Cov_{Q*}\left({\hat{\gamma }}_{i},{\hat{\gamma }}_{j}\right){\hat{\alpha }}_{j}\right]\\ & = & \sum_{i=1}^{\left(q+1\right)}\sum_{j=1}^{\left(q+1\right)}Cov_{Q*}\left({\hat{\gamma }}_{i},{\hat{\gamma }}_{j}\right)E_{G}\left[{\hat{\alpha }}_{i}{\hat{\alpha }}_{j}\right]\\ & = & \sum_{i=1}^{\left(q+1\right)}\sum_{j=1}^{\left(q+1\right)}Cov_{Q*}\left({\hat{\gamma }}_{i},{\hat{\gamma }}_{j}\right)\left(\alpha_{i}\alpha_{j}+Cov\left({\hat{\alpha }}_{i},{\hat{\alpha }}_{j}\right)\right)\\ & = & \sum_{i=1}^{\left(q+1\right)}\sum_{j=1}^{\left(q+1\right)}Cov_{Q*}\left({\hat{\gamma }}_{i},{\hat{\gamma }}_{j}\right)\alpha_{i}\alpha_{j}+\sum_{i=1}^{\left(q+1\right)}\sum_{j=1}^{\left(q+1\right)}Cov_{Q*}\left({\hat{\gamma }}_{i},{\hat{\gamma }}_{j}\right)Cov\left({\hat{\alpha }}_{i},{\hat{\alpha }}_{j}\right)\\ & = & \alpha^{T}Cov_{Q*}\left(\hat{\Gamma *}\right)\alpha +Tr\left[Cov_{Q*}\left(\hat{\Gamma *}\right)Cov_{G}\left(\hat{\alpha }\right)\right] \end{array}$$

However, $Cov_{G}\left(\hat{\alpha }\right)$ in Eq. (6) emanates from several interacting models, and in order to present the variance formula in a format that would later on allow estimation of, $Cov_{G}\left(\hat{\alpha }\right)$ is further decomposed using the law of total covariance:

(7) $$\begin{array}{ccc} Cov_{G}\left(\hat{\alpha }\right) & = & Cov_{G}\left(\hat{A*}\hat{\beta }\right)\\ & = & E_{F}\left[Cov_{G*}\left(\hat{A*}\hat{\beta }\right)\right]+Cov_{F}\left(E_{G*}\left[\hat{A*}\hat{\beta }\right]\right)\\ & = & E_{F}\left[{\hat{\beta }}^{T}Cov_{G*}\left(\hat{A*}\right)\hat{\beta }\right]+A*Cov_{F}\left(\hat{\beta }\right)A{*}^{T}\\ & = & \beta^{T}Cov_{G*}\left(\hat{A*}\right)\beta +A*Cov_{F}\left(\hat{\beta }\right)A{*}^{T}+Tr\left[Cov_{G*}\left(\hat{A*}\right)Cov_{F}\left(\hat{\beta }\right)\right], \end{array}$$

where the third term ($Tr[Cov_{G^{*}}\left(\hat{A^{*}}\right)Cov_{F}\left(\hat{\beta }\right)]$) follows from $E_{F}\left[{\hat{\beta }}^{T}\hat{\beta }\right]=\beta^{T}\beta +Cov\left(\hat{\beta }\right)$.

Thus, the variance of the AGBD predictor can be expressed as:

(8a) $$\begin{array}{ccc} V\left({\hat{\bar{y}}}_{U_{3pHMB}}\right) & = & {\bar{p}}_{U}\left(\alpha^{T}Cov_{Q*}\left(\hat{\Gamma *}\right)\alpha \right){\bar{p}}_{U}^{T}\\ & & +\;{\bar{p}}_{U}\left(\Gamma *\beta^{T}Cov_{G*}\left(\hat{A*}\right)\beta \;\Gamma {*}^{T}\right){\bar{p}}_{U}^{T}\\ & & +\;{\bar{p}}_{U}\left(\Gamma *A*Cov_{F}\left(\hat{\beta }\right)A{*}^{T}\;\Gamma {*}^{T}\right){\bar{p}}_{U}^{T}\\ & & +\;{\bar{p}}_{U}\;\left(\Gamma *Tr\left[Cov_{G*}\left(\hat{A*}\right)Cov_{F}\left(\hat{\beta }\right)\right]\;\Gamma {*}^{T}\right){\bar{p}}_{U}^{T}\\ & & +\;{\bar{p}}_{U}\left(Tr\left[Cov_{Q*}\left(\hat{\Gamma *}\right)\left(\beta^{T}Cov_{G*}\left(\hat{A*}\right)\beta +A*Cov_{F}\left(\hat{\beta }\right)A{*}^{T}+Tr\left[Cov_{G*}\left(\hat{A*}\right)Cov_{F}\left(\hat{\beta }\right)\right]\right)\right]\right){\bar{p}}_{U}^{T}. \end{array}$$

In the simulation analyses that we performed to validate our methodology (illustrated in Table 7), we found that all the terms involving traces were small, and could be neglected in the variance formula, for simplification. Thus, a good approximation of the variance of the AGBD predictor would be

(8b) $$V\left({\hat{\bar{y}}}_{U3pHMB}\right)={\bar{p}}_{U}\left(\alpha^{T}Cov_{Q^{*}}\left(\hat{\Gamma^{*}}\right)\alpha \right){\bar{p}}_{U}^{T}+{\bar{p}}_{U}\left(\Gamma^{*}\beta^{T}Cov_{G^{*}}\left(\hat{A^{*}}\right)\beta \;\Gamma^{{*}^{T}}\right){\bar{p}}_{U}^{T}+{\bar{p}}_{U}\left(\Gamma^{*}A^{*}Cov_{F}\left(\hat{\beta }\right)A^{{*}^{T}}\;\Gamma^{{*}^{T}}\right){\bar{p}}_{U}^{T}.$$

By replacing the covariances of estimated model parameters and the model parameters by their corresponding estimators, we obtain a variance estimator. In the simulation analysis that we performed to validate our methodology, it was found that the variance estimators were approximately unbiased.

If we would like to derive the MSE of the predictor rather than the variance, we may define the true AGBD over the AOI through the models involved. We first express the AGBD variable using the model Q as

$$Z_{U}\alpha =P_{U}\gamma +e_{U},$$

Using model G, we can express $Z_{U}\alpha$ as $X_{U}\beta -\upsilon_{U}$ and thus $X_{U}\beta -\upsilon_{U}=P_{U}\gamma +e_{U}$, leading to

$$X_{U}\beta =P_{U}\gamma +u_{U}+\upsilon_{U}.$$

Using model F, we can express $X_{U}\beta$ as $y_{U}-\epsilon_{U}$ and thus $y_{U}-\epsilon_{U}=P_{U}\gamma +u_{U}+\upsilon_{U}$, which implies that the variable of interest for all elements in the AOI can be expressed using models F, G and Q as

$$y_{U}=P_{U}\gamma +e_{U}+\upsilon_{U}+\epsilon_{U}.$$

The AGBD mean for U can thus be expressed as

$${\bar{y}}_{U}=\frac{1}{N}\sum_{i=1}^{N}\left(p_{i}\gamma +e_{i}+\upsilon_{i}+\epsilon_{i}\right)={\bar{p}}_{U}\gamma +{\overset{‾}{e}}_{U}+{\overset{‾}{\upsilon }}_{U}+{\overset{‾}{\epsilon }}_{U}.$$

The MSE of the predictor ${\hat{\bar{y}}}_{U_{3pHMB}}$ is then, according to the definition of MSE,

$$\begin{array}{ccc} MSE\left({\hat{\bar{y}}}_{U_{3pHMB}}\right) & = & E\left[{\left({\hat{\bar{y}}}_{U_{3pHMB}}-{\bar{y}}_{U}\right)}^{2}\right]\\ & = & E\left[{\left({\bar{p}}_{U}\hat{\gamma }-{\bar{p}}_{U}\gamma -{\bar{e}}_{U}-{\bar{\upsilon }}_{U}-{\bar{\in }}_{U}\right)}^{2}\right]. \end{array}$$

Under an assumption of independence between the variability terms, the MSE can be expressed as

(9) $$MSE\left({\hat{\bar{y}}}_{U3pHMB}\right)={\bar{p}}_{U}Cov\left(\hat{\gamma }\right){\bar{p}}_{U}^{T}+\frac{\theta^{2}}{N}+\frac{\delta^{2}}{N}+\frac{\omega^{2}}{N}=V\left({\hat{\bar{y}}}_{U3pHMB}\right)+\frac{\theta^{2}}{N}+\frac{\delta^{2}}{N}+\frac{\omega^{2}}{N}.$$

However, note that it is not obvious that the three variability terms are independent, and typically there is also spatial autocorrelation among them. Thus, MSE expressions become complicated and require further investigation.

### Details of three-phase hierarchical hybrid inference (3pHHY)

Next, we present our second case, i.e., when the last step auxiliary information is available only for a probability sample from the AOI, rather than wall-to-wall. This step is the only difference compared to 3pHMB inference. In our forest inventory example, the auxiliary information in this case could be retrieved from the ICESat-2 spaceborne LiDAR.

Thus, ${\bar{p}}_{U}$ in this case is estimated using the design-based estimator (e.g., [30], p. 42)

$${\hat{\bar{p}}}_{U}=\frac{1}{N}\sum_{S_{IV}}\frac{p_{k}}{\pi_{k}},$$

where $S_{IV}$ is a probability sample drawn from the AOI, $p_{k}$ is a vector of ICESat-2 auxiliary values for the $k^{th}$ element in the sample, and $\pi_{k}$ is the probability of including the $k^{th}$ element to the sample $S_{IV}$.

The design-based expectation of ${\hat{\bar{p}}}_{U}$ is

$$E_{D}\left[{\hat{\bar{p}}}_{U}\right]={\bar{p}}_{U}.$$

The 3pHHY predictor of the AGBD mean over the AOI is then (cf., [31], [32], [33])

(10) $${\hat{\bar{y}}}_{U3pHHY}=\frac{1}{N}\sum_{S_{IV}}\frac{p_{k}}{\pi_{k}}\hat{\gamma }.$$

**Fig. 2:**
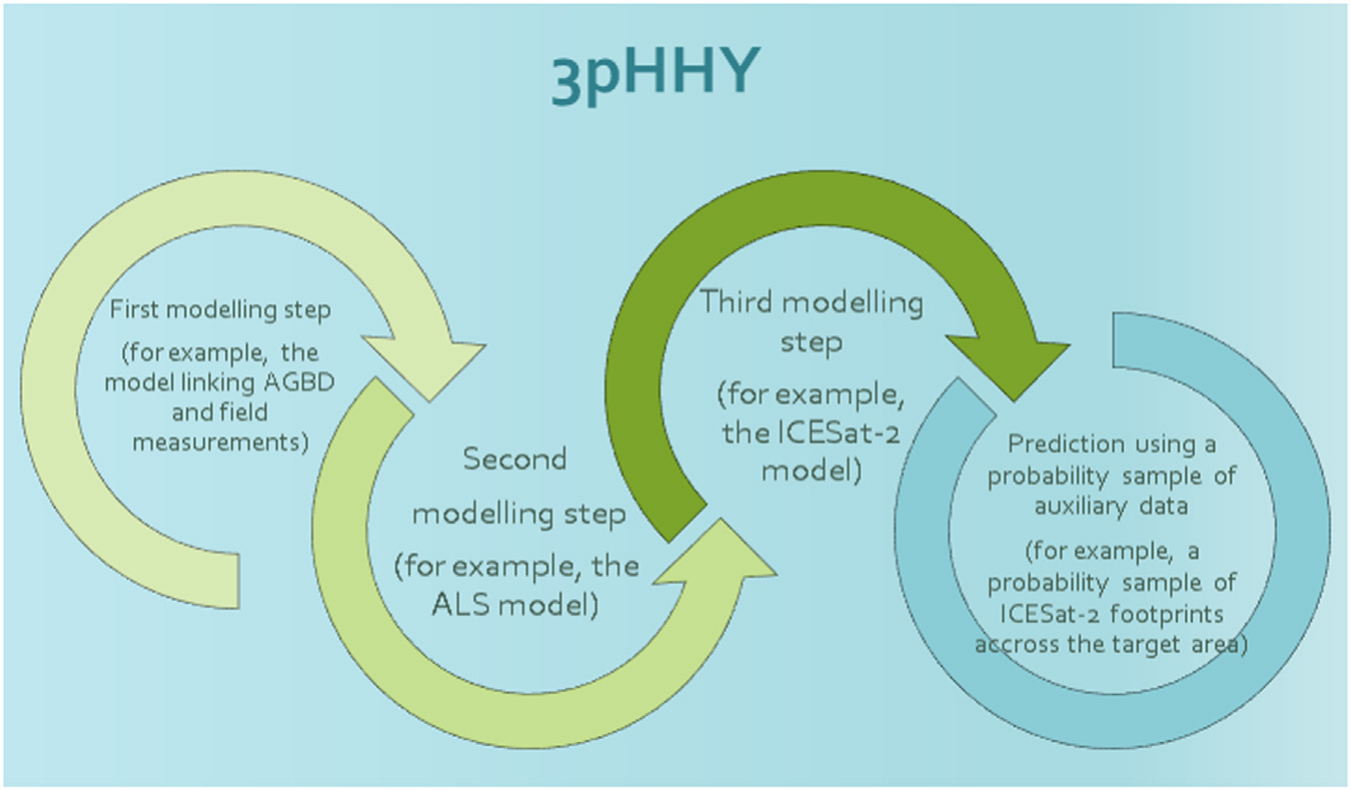
A graphical overview on the 3pHHY prediction based on a probability sample of auxiliary (remotely sensed) data.

Fig. 2 gives a graphical overview of 3pHHY prediction.

### The 3pHHY predictor variance and its estimator

To derive the variance of the 3pHHY predictor, we begin with the expectation of the predictor, which can be decomposed as

$$\begin{array}{ccc} E\left[{\hat{\bar{y}}}_{U3pHHY}\right] & = & E\left[\frac{1}{N}\sum_{S_{IV}}\frac{p_{k}}{\pi_{k}}\hat{\gamma }\right]\;=E_{D}\left[E_{Q}\left[\frac{1}{N}\sum_{S_{IV}}\frac{p_{k}}{\pi_{k}}\hat{\gamma }\right]\right]\\ & = & E_{D}\left[\frac{1}{N}\sum_{S_{IV}}\frac{p_{k}}{\pi_{k}}E_{Q}\left[\hat{\gamma }\right]\right]\;={\bar{p}}_{U}E_{Q}\left[\hat{\gamma }\right]={\bar{p}}_{U}\Gamma^{*}A^{*}\beta ={\bar{p}}_{U}\gamma , \end{array}$$

where the subscript D denotes expectation due to the design.

The variance of the 3pHHY predictor can be decomposed using the law of total variance as

(11) $$\begin{array}{ccc} V\left({\hat{\bar{y}}}_{U3pHHY}\right) & = & V\left(\frac{1}{N}\sum_{S_{IV}}\frac{p_{k}}{\pi_{k}}\hat{\gamma }\right)\\ & = & V_{D}\left(E_{Q}\left[\frac{1}{N}\sum_{S_{IV}}\frac{p_{k}}{\pi_{k}}\hat{\gamma }\right]\right)+E_{D}\left[V_{Q}\left(\frac{1}{N}\sum_{S_{IV}}\frac{p_{k}}{\pi_{k}}\hat{\gamma }\right)\right]\\ & = & V_{D}\left(\frac{1}{N}\sum_{S_{IV}}\frac{p_{k}}{\pi_{k}}\gamma \right)+E_{D}\left[\frac{1}{N}\sum_{S_{IV}}\frac{p_{k}}{\pi_{k}}Cov\left(\gamma \right){\left(\frac{1}{N}\sum_{S_{IV}}\frac{p_{k}}{\pi_{k}}\right)}^{T}\right]\\ & = & \gamma^{T}Cov\left(\frac{1}{N}\sum_{S_{IV}}\frac{p_{k}}{\pi_{k}}\right)\gamma +{\bar{p}}_{U}Cov\left(\hat{\gamma }\right){\bar{p}}_{U}^{T}+Tr\left[Cov\left(\frac{1}{N}\sum_{S_{IV}}\frac{p_{k}}{\pi_{k}}\right)Cov\left(\hat{\gamma }\right)\right]. \end{array}$$

The third term in the final expression of Eq. (11) is due to

$$E_{D}\left[\frac{1}{N}\sum_{S_{IV}}\frac{p_{k}}{\pi_{k}}{\left(\frac{1}{N}\sum_{S_{IV}}\frac{p_{k}}{\pi_{k}}\right)}^{T}\right]={\bar{p}}_{U}{\bar{p}}_{U}^{T}+Cov\left(\frac{1}{N}\sum_{S_{IV}}\frac{p_{k}}{\pi_{k}}\right).$$

Our simulation analysis showed that the third term, $Tr[Cov\left(\frac{1}{N}\sum_{S_{IV}}\frac{p_{k}}{\pi_{k}}\right)Cov\left(\hat{\gamma }\right)]$, in Eq. (11) is small and may be neglected. Thus, for any given sampling design the 3pHHY variance is

(12) $$V\left({\hat{\bar{y}}}_{U3pHHY}\right)=\gamma^{T}Cov\left(\frac{1}{N}\sum_{S_{IV}}\frac{p_{k}}{\pi_{k}}\right)\gamma +{\bar{p}}_{U}Cov\left(\hat{\gamma }\right){\bar{p}}_{U}^{T}.$$

Under a simple random sampling without replacement design, the variance is

(13) $$V\left({\hat{\bar{y}}}_{U3pHHY}\right)=\frac{1}{n_{IV}}\left(1-\frac{n_{IV}}{N}\right)\gamma^{T}Cov\left(P_{U}\right)\gamma +{\bar{p}}_{U}Cov\left(\hat{\gamma }\right){\bar{p}}_{U}^{T},$$

where $n_{IV}$ is the sample size, $\left(1-\frac{n_{IV}}{N}\right)$ is the finite population correction factor, and $Cov(P_{U})$ is the population covariance of the ICESat-2 explanatory variables. In the simulations performed to validate the results, Eq. (13) was used, since simple random sampling was assumed.

By replacing the covariance of estimated model parameters, $Cov(\hat{\gamma })$, the model parameters, γ, and the design-based covariance $Cov(\frac{1}{N}\sum_{S_{IV}}\frac{p_{k}}{\pi_{k}})$ with their corresponding estimators we obtain a variance estimator. Our simulation analysis showed that the variance estimator is approximately unbiased.

If we would like to derive the MSE of the 3pHHY predictor, we may proceed along the route previously outlined for 3pHMB, i.e.

(14) $$\begin{array}{ccc} MSE\left({\hat{\bar{y}}}_{U3pHHY}\right) & = & E\left[{\left({\hat{\bar{y}}}_{U3pHHY}-{\bar{y}}_{U}\right)}^{2}\right]\\ & = & E\left[{\left(\frac{1}{N}\sum_{S_{IV}}\frac{p_{k}}{\pi_{k}}\hat{\gamma }-{\bar{p}}_{U}\gamma -{\bar{e}}_{U}-{\bar{\upsilon }}_{U}-{\bar{\in }}_{U}\right)}^{2}\right]\\ & = & E\left[{\left(\frac{1}{N}\sum_{S_{IV}}\frac{p_{k}}{\pi_{k}}\hat{\gamma }-{\bar{p}}_{U}\hat{\gamma }+\;{\bar{p}}_{U}\hat{\gamma }-{\bar{p}}_{U}\gamma -{\bar{e}}_{U}-{\bar{\upsilon }}_{U}-{\bar{\in }}_{U}\right)}^{2}\right]\\ & = & E\left[{\left(\left(\frac{1}{N}\sum_{S_{IV}}\frac{p_{k}}{\pi_{k}}-{\bar{p}}_{U}\right)\hat{\gamma }+\;{\bar{p}}_{U}\left(\hat{\gamma }-\gamma \right)-{\bar{e}}_{U}-{\bar{\upsilon }}_{U}-{\bar{\in }}_{U}\right)}^{2}\right]\\ & = & \gamma^{T}Cov\left(\frac{1}{N}\sum_{S_{IV}}\frac{p_{k}}{\pi_{k}}\right)\gamma +{\bar{p}}_{U}Cov\left(\hat{\gamma }\right){\bar{p}}_{U}^{T}+Tr\left[Cov\left(\frac{1}{N}\sum_{S_{IV}}\frac{p_{k}}{\pi_{k}}\right)Cov\left(\hat{\gamma }\right)\right]+\frac{\theta^{2}}{N}+\frac{\delta^{2}}{N}+\frac{\omega^{2}}{N}\\ & = & V\left({\hat{\bar{y}}}_{U3pHHY}\right)+\frac{\theta^{2}}{N}+\frac{\delta^{2}}{N}+\frac{\omega^{2}}{N}. \end{array}$$

Like in the case of 3pHMB, this MSE formula assumes independence between the variability terms and absence of spatial autocorrelation. Thus, deriving a useful MSE expression is complicated and requires further investigation.

### Study of hierarchical modeling in the context of a superpopulation model

The previous sections of this article have provided the formulas needed for applying 3pHMB and 3pHHY for prediction, and for assessing uncertainties. In this section, we study properties of hierarchical modeling that cannot be straightforwardly deduced from the formulas presented in the previous sections. We do this by specifying a superpopulation model with normally distributed random variables. Some of the non-standard models from the previous sections, e.g., Eqs. (2) and (3), will now be derived through conditional expectation, thus validating the previously presented models. This section will also provide insights into effects of hierarchical modeling that are important to understand, such as the reduced variability of the response variable the more hierarchically nested models are added. Examples of the magnitude of such variability reduction are provided.

We assume that with each population element a multivariate random variable is associated, i.e., for the $i^{th}$ observation, the values ($y_{i}$, $x_{i}$, $z_{i}$, $p_{i}$) are outcomes from a multivariate random variable ($Y_{i}$, $X_{i}$, $Z_{i}$, $P_{i}$). The joint distribution of the random variables for the elements in the population defines a superpopulation model denoted ξ ([34], p. 80).

The superpopulation model ξ shapes the relationship between the variables in the population, and the observations linked to them. We assume the same type of population elements, variables and observations as previously, i.e. $y_{i}$ is the AGBD measured in the field, $x_{i}$ is some field plot measurements, such as average tree diameter (DBH), $z_{i}$ is some ALS variable, and $p_{i}$ some Landsat (in the case of 3pHMB) or ICESat-2 variable (in the case of 3pHHY). The values ($y_{i}$, $x_{i}$, $z_{i}$, $p_{i}$) are realizations from the corresponding multivariate random variable ($Y_{i}$, $X_{i}$, $Z_{i}$, $P_{i}$).

We assume a multivariate normal distribution, i.e.

$$\left(\begin{array}{c} Y_{i}\\ X_{i}\\ \begin{array}{c} Z_{i}\\ P_{i} \end{array} \end{array}\right)∼N\left(\mu ,\Sigma \right),$$

where $\mu =(\mu_{Y},\mu_{X},\mu_{Z},\mu_{P})$ is a vector of superpopulation means, and Σ is the superpopulation variance-covariance matrix with variances $\sigma_{\left(\cdot \right)}^{2}$ for each variable on the diagonal and covariances $\sigma_{\left(\cdot )(\cdot \right)}$ between the variables on the off-diagonal elements. We assume that our multivariate variables are exchangeable, i.e., every permutation of the order of the population elements follows the same joint distribution ξ ([34], p. 85).

We now derive the models previously presented Eqs. (1), ((2), and (3)) through the superpopulation model. We begin with describing the model linking AGBD and DBH, i.e., the model F. Following the joint distribution ξ, the conditional probability density function of $Y_{i}$, given that $X_{i}=x_{i}$ for the $i^{th}$ unit, is

$$f_{Y|X}\left(y|x\right)=\;\frac{f_{X,Y}\left(x,y\right)}{f_{X}\left(x\right)},$$

where $f_{X,Y}\left(x,y\right)$ is the joint probability density function of Y and X, and $f_{X}\left(x\right)$ is the marginal density function for X. Under the normality assumption the conditional expectation of $Y_{i}$ given $X_{i}=x_{i}$ can be expressed as ([35], p. 72)

(15) $$E\left[Y_{i}|X_{i}=x_{i}\right]=E\left[Y_{i}\right]+\frac{Cov\left(Y_{i},X_{i}\right)}{V\left(X_{i}\right)}\left(x_{i}-E\left[X_{i}\right]\right)=\mu_{Y}+r_{YX}\frac{\sigma_{Y}}{\sigma_{X}}\left(x_{i}-\mu_{X}\right)=\mu_{Y|X}$$

where $r_{YX}$ is the correlation between $Y_{i}$ and $X_{i}$, and $\sigma_{\left(\cdot \right)}$ is the superpopulation standard deviation for the given variable. The variance of the conditional distribution is ([35], p. 72)

(16) $$Var\left(Y_{i}|X_{i}=x_{i}\right)=V\left(Y_{i}\right)-\frac{Cov^{2}\left(Y_{i},X_{i}\right)}{V\left(X_{i}\right)}=\sigma_{Y}^{2}\left(1-r_{YX}^{2}\right)=\sigma_{Y|X}^{2}.$$

The conditional distribution of $Y_{i}$, given that $X_{i}=x_{i}$ for the $i^{th}$ observation is then

$$Y_{i}|X_{i}=x_{i}∼N\left(\mu_{Y|X},\;\sigma_{Y|X}^{2}\right).$$

Thus, in our example, the AGBD random variable associated with the $i^{th}$ population element has the expectation $\mu_{Y}$ and variance $\sigma_{Y}^{2}$; $X_{i}$ is the DBH random variable with expectation $\mu_{X}$ and variance $\sigma_{X}^{2}$. However, for a fixed outcome $x_{i}$ from $X_{i}$, there is a random subset of $Y_{i}$ conditional on the fixed $x_{i}$; this subset is the variable $Y_{i}|X_{i}=x_{i}$ with expectation $\mu_{Y|X}=\mu_{Y}+r_{YX}\frac{\sigma_{Y}}{\sigma_{X}}\left(x_{i}-\mu_{X}\right)$ and variance $\sigma_{Y|X}^{2}=\sigma_{Y}^{2}\left(1-r_{YX}^{2}\right)$. This corresponds to regressing $Y_{i}$ on $X_{i}$, which reveals how much information about $Y_{i}$ is contained in an observation of $X_{i}$ ([35], p. 73).

Therefore, using the conditional distribution, we can define our first model (F), which links AGBD and DBH as

(17) $$F:\;Y_{i}=\mu_{Y|X}+\epsilon_{i},\;\epsilon_{i}∼N\left(0,\sigma_{Y|X}^{2}\right),$$

where $\epsilon_{i}$ is the variability term. By denoting $\omega^{2}=\sigma_{Y|X}^{2}$, $\beta_{0}=\left(\mu_{Y}-r_{YX}\frac{\sigma_{Y}}{\sigma_{X}}\mu_{X}\right)$ and $\beta_{1}=r_{YX}\frac{\sigma_{Y}}{\sigma_{X}}$ we can rewrite F in the form previously introduced, i.e.

$$F:\;Y_{i}=\beta_{0}+\beta_{1}x_{i}+\epsilon_{i},\;\epsilon_{i}∼N\left(0,\omega^{2}\right),$$

and in linear algebra notation

$$F:\;Y_{i}=x_{i}\beta +\epsilon_{i},\;\epsilon_{i}∼N\left(0,\omega^{2}\right),$$

where $\beta ={\left(\beta_{0},\beta_{1}\right)}^{T}$ and $x_{i}=\left(1,x_{i}\right)$. By substituting the variances and correlations in the parameter formulas with their corresponding estimators, we obtain the well-known formulas for parameter estimation from OLS regression.

Similarly, to derive the model G (Eq. (2)), we introduce $E[Y_{i}|X_{i}]$, which is a random variable since we now condition on the random variable $X_{i}$ rather than some fixed outcome $X_{i}=x_{i}$ ([27], p. 131–132). The variance of $E[Y_{i}|X_{i}]$ is

(18) $$V\left(E\left[Y_{i}|X_{i}\right]\right)=V\left(\mu_{Y}+r_{YX}\frac{\sigma_{Y}}{\sigma_{X}}\left(X_{i}-\mu_{X}\right)\right)=V\left(r_{YX}\frac{\sigma_{Y}}{\sigma_{X}}X_{i}\right)=r_{YX}^{2}\frac{\sigma_{Y}^{2}}{\sigma_{X}^{2}}V\left(X_{i}\right)=r_{YX}^{2}\frac{\sigma_{Y}^{2}}{\sigma_{X}^{2}}\sigma_{X}^{2}=r_{YX}^{2}\sigma_{Y}^{2}.$$

The expectation of $E[Y_{i}|X_{i}]$ can be derived through the law of iterated expectations ([27], p. 132)

(19) $$E\left[E\left[Y_{i}|X_{i}\right]\right]=E\left[Y_{i}\right]=\mu_{Y}.$$

The next step is to introduce the ALS variable, $Z_{i}$. Based on our superpopulation model, the conditional distribution of $E[Y_{i}|X_{i}]$ given that $Z_{i}=z_{i}$ has the expected value (cf. Eq. (15))

(20) $$\begin{array}{ccc} E\left[E\left[Y_{i}|X_{i}\right]|Z_{i}=z_{i}\right] & = & E\left[E\left[Y_{i}|X_{i}\right]\right]+\frac{Cov\left(E\left[Y_{i}|X_{i}\right],Z_{i}\right)}{V\left(Z_{i}\right)}\left(z_{i}-E\left[Z_{i}\right]\right)\\ & = & \mu_{Y}+r_{XZ}r_{YX}\frac{\sigma_{Y}}{\sigma_{Z}}\left(z_{i}-\mu_{z}\right)\;=\mu_{Y\left|X\right|Z}, \end{array}$$

where,

$$\begin{array}{ccc} Cov\left(E\left[Y_{i}|X_{i}\right],Z_{i}\right) & = & Cov\left(\left(\mu_{Y}+r_{YX}\frac{\sigma_{Y}}{\sigma_{X}}\left(X_{i}-\mu_{X}\right)\right),Z_{i}\right)\\ & = & Cov\left(r_{YX}\frac{\sigma_{Y}}{\sigma_{X}}X_{i},\;Z_{i}\right)\;=r_{YX}\frac{\sigma_{Y}}{\sigma_{X}}Cov\left(X_{i},Z_{i}\right)\\ & = & r_{YX}\frac{\sigma_{Y}}{\sigma_{X}}r_{XZ}\sqrt{V\left(X_{i}\right)V\left(Z_{i}\right)}\;=r_{XZ}r_{YX}\sigma_{Y}\sigma_{Z}. \end{array}$$

The variance is (cf. Eq. (16))

(21) $$\begin{array}{ccc} Var\left(E\left[Y_{i}|X_{i}\right]|Z_{i}=z_{i}\right) & = & V\left(E\left[Y_{i}|X_{i}\right]\right)-\frac{Cov^{2}\left(E\left[Y_{i}|X_{i}\right],Z_{i}\right)}{V\left(Z_{i}\right)}\\ & = & r_{YX}^{2}\sigma_{Y}^{2}-\frac{{\left(r_{XZ}r_{YX}\sigma_{Y}\sigma_{Z}\right)}^{2}}{\sigma_{Z}^{2}}\;=\sigma_{Y}^{2}r_{YX}^{2}\left(1-r_{XZ}^{2}\right)\;=\sigma_{Y|X|Z}^{2} \end{array}$$

The conditional distribution of $E[Y_{i}|X_{i}]$, given that $Z_{i}=z_{i}$ is then

$$E\left[Y_{i}|X_{i}\right]|Z_{i}=z_{i}∼N\left(\mu_{Y\left|X\right|Z},\;\sigma_{Y\left|X\right|Z}^{2}\right).$$

Thus, we can define the model G:

(22) $$G:\;E\left[Y_{i}|X_{i}\right]=\mu_{Y\left|X\right|Z}+\upsilon_{i},\;\upsilon_{i}∼N\left(0,\sigma_{Y\left|X\right|Z}^{2}\right).$$

Denoting model parameters $\alpha_{0}=\left(\mu_{Y}-r_{XZ}r_{YX}\frac{\sigma_{Y}}{\sigma_{Z}}\mu_{Z}\right)$, $\alpha_{1}=r_{XZ}r_{YX}\frac{\sigma_{Y}}{\sigma_{Z}}$, and the variance of the variability term $\upsilon_{i}$ as $\delta^{2}=\sigma_{Y|X|Z}^{2}$, we can rewrite G as

$$G:\;E\left[Y_{i}|X_{i}\right]=\alpha_{0}+\alpha_{1}z_{i}+\upsilon_{i},\;\upsilon_{i}∼N\left(0,\delta^{2}\right)$$

and in linear algebra notation

$$G:\;X_{i}\beta =z_{i}\alpha +\upsilon_{i},\;\upsilon_{i}∼N\left(0,\delta^{2}\right),$$

where $X_{i}=\left(1,\;X_{i}\right)$, $\alpha ={\left(\alpha_{0},\;\alpha_{1}\right)}^{T}$ and $z_{i}=\left(1,\;z_{i}\right)$. This model coincides with the model presented in Eq. (2) in the previous section.

It remains to derive the model Q, (Eq. (3)) in a similar fashion. Thus, we define a new random variable, $E[E\left[Y_{i}|X_{i}\right]|Z_{i}]$, which has variance

(23) $$V\left(E\left[E\left[Y_{i}|X_{i}\right]|Z_{i}\right]\right)=\;V\left(\mu_{Y}-r_{XZ}r_{YX}\frac{\sigma_{Y}}{\sigma_{Z}}\left(Z_{i}-\mu_{Z}\right)\right)=V\left(r_{XZ}r_{YX}\frac{\sigma_{Y}}{\sigma_{Z}}Z_{i}\right)=r_{XZ}^{2}r_{YX}^{2}\sigma_{Y}^{2},$$

and expectation (following the law of iterated expectations)

(24) $$E\left[E\left[E\left[Y_{i}|X_{i}\right]|Z_{i}\right]\right]=E\left[Y_{i}\right]=\mu_{Y}.$$

The conditional distribution of $E[E\left[Y_{i}|X_{i}\right]|Z_{i}]$ given $P_{i}=p_{i}$ is

$$E\left[E\left[Y_{i}|X_{i}\right]|Z_{i}\right]|P_{i}=p_{i}∼N\left(\mu_{Y\left|X\right|Z|P},\;\sigma_{Y\left|X\right|Z|P}^{2}\right),$$

where

(25) $$E\left[E\left[E\left[Y_{i}|X_{i}\right]|Z_{i}\right]|P_{i}=p_{i}\right]=\mu_{Y}+r_{ZP}r_{XZ}r_{YX}\frac{\sigma_{Y}}{\sigma_{P}}\left(p_{i}-\mu_{P}\right)=\mu_{Y\left|X\right|Z|P},$$

given that the covariance

$$Cov\left(\left(E\left[E\left[Y_{i}|X_{i}\right]|Z_{i}\right]\right),P_{i}\right)=Cov\left(\left(\mu_{Y}-r_{XZ}r_{YX}\frac{\sigma_{Y}}{\sigma_{Z}}\left(Z_{i}-\mu_{Z}\right)\right),P_{i}\right)\;=r_{XZ}r_{YX}\frac{\sigma_{Y}}{\sigma_{Z}}Cov\left(Z_{i},P_{i}\right)\;=r_{XZ}r_{YX}\sigma_{Y}\sigma_{P},$$

and the variance is

(26) $$V\left(E\left[E\left[Y_{i}|X_{i}\right]|Z_{i}\right]|P_{i}=p_{i}\right)\;=\sigma_{Y}^{2}r_{YX}^{2}r_{XZ}^{2}\left(1-r_{ZP}^{2}\right)=\sigma_{Y\left|X\right|Z|P}^{2}.$$

Thus, the model Q is

(27) $$Q:\;E\left[E\left[Y_{i}|X_{i}\right]|Z_{i}\right]=\mu_{Y\left|X\right|Z|P}+e_{i},\;e_{i}∼N\left(0,\sigma_{Y\left|X\right|Z|P}^{2}\right).$$

Denoting the model parameters as $\gamma_{0}=\left(\mu_{Y}-r_{ZP}r_{XZ}r_{YX}\frac{\sigma_{Y}}{\sigma_{P}}\mu_{P}\right)$ and $\gamma_{1}=r_{ZP}r_{XZ}r_{YX}\frac{\sigma_{Y}}{\sigma_{P}}$, the variance of the variability term $e_{i}$ as $\theta^{2}=\sigma_{Y|X|Z|P}^{2}$, we can rewrite Q as

$$Q:\;E\left[E\left[Y_{i}|X_{i}\right]|Z_{i}\right]=\gamma_{0}+\gamma_{1}p_{i}+e_{i},\;e_{i}∼N\left(0,\theta^{2}\right),$$

and in linear algebra notation as

$$Q:\;Z_{i}\alpha =p_{i}\gamma +e_{i},\;e_{i}∼N\left(0,\theta^{2}\right),$$

where $Z_{i}=\left(1,\;Z_{i}\right)$, $\gamma ={\left(\gamma_{0},\;\gamma_{1}\right)}^{T}$ and $p_{i}=\left(1,\;p_{i}\right)$, which coincides with Eq. (3) in the previous section.

### Decreased variability of the response variable

Deriving the models through conditional expectation reveals properties of 3pHMB (and 3pHHY) that cannot be immediately observed from the first section of this article. An important property to note is that as more modeling steps are included, the variability of the response variable decreases. This decreased variability (of proxy AGBD in our example) may have important implications in applications. One obvious case is if the objective is to map the AGBD distribution across a landscape. In case several hierarchically nested models have been utilized, the mapped AGBD variability in the landscape would be substantially smaller than the real variability. A potential solution to avoiding such decreased variability could be to apply calibration (e.g., [36]).

The decreased variability may also have negative implications if the objective is to predict the AGBD, not least for domains. As pointed out by Chambers and Clark [37], a means to minimize the uncertainty of model-based predictors is to ensure first-order balanced samples, meaning that the mean values of explanatory variables in the dataset used for model estimation coincide with the mean values of the explanatory variables in the target population. Although not specifically studied in this article, we hypothesize that first-order balanced sampling becomes increasingly important the more modeling steps are included. Further, whereas the most relevant uncertainty measure in connection with model-based inference would be the MSE, several studies suggest that for large areas, the relative difference between MSE and variance would be small (e.g., [33], [38]) in case model-unbiased predictors are applied. However, in case the variability of the response variable is substantially reduced, it remains to be evaluated if this assertion holds.

Thus, model-based inference should be applied with caution in case the variability of the (proxy) response variable is substantially reduced compared to the real-world variability of the response variable. Below, we demonstrate how the variance of the response variable is affected by multi-step modeling (the models F, G and Q).

Table 2 gives an overview of the models and the corresponding expectation and variance of the response variable, under the previously introduced superpopulation model.

**Table 2:** The expectation and variance of the response variable used in models F, G and Q.

Model	Response variable	Expectation of the response variable	Variance of the response variable
F	$Y_{i}$	$\mu_{Y}$	$\sigma_{Y}^{2}$
G	$E[Y_{i}|X_{i}]$	$\mu_{Y}$	$r_{YX}^{2}\sigma_{Y}^{2}$
Q	$E[E\left[Y_{i}|X_{i}\right]|Z_{i}]$	$\mu_{Y}$	$r_{XZ}^{2}r_{YX}^{2}\sigma_{Y}^{2}$

From Table 2, we can see that whereas the response variables have the same expectation, their variability around the expectation is not the same. With the increased hierarchy of conditions, the variability of the proxy target variable is decreasing in comparison to the variability of the actual target variable.

In Tables 3 and 4 we show numerical examples of the reduction of the variance of the response variable. In Table 3, this is shown for model G, i.e., for the case of two modeling steps.

**Table 3:** The decrease of the variance of the response variable in model G ($r_{YX}^{2}\sigma_{Y}^{2}$) for different correlations ($r_{YX}$) between the explanatory and response variables in model F.

=0.9	$r_{YX}=0.8$	$r_{YX}=0.7$	$r_{YX}=0.6$
$0.81\;\times \;\sigma_{Y}^{2}$	$0.64\;\times \sigma_{Y}^{2}$	$0.49\;\times \sigma_{Y}^{2}$	$0.36\;\times \sigma_{Y}^{2}$

**Table 4:** The variance of the response variable for model Q ($r_{XZ}^{2}r_{YX}^{2}\sigma_{Y}^{2}$) for different correlations between the explanatory and response variables in model F and model G ($r_{YX}$ and $r_{XZ}$).

	$r_{YX}=0.9$	$r_{YX}=0.8$	$r_{YX}=0.7$	$r_{YX}=0.6$
$r_{XZ}=0.9$	$0.66\;\times \sigma_{Y}^{2}$	$0.52\;\times \sigma_{Y}^{2}$	$0.40\;\times \sigma_{Y}^{2}$	$0.29\;\times \sigma_{Y}^{2}$
$r_{XZ}=0.8$	$0.52\;\times \sigma_{Y}^{2}$	$0.41\;\times \sigma_{Y}^{2}$	$0.31\;\times \sigma_{Y}^{2}$	$0.23\;\times \sigma_{Y}^{2}$
$r_{XZ}=0.7$	$0.40\;\times \sigma_{Y}^{2}$	$0.31\;\times \sigma_{Y}^{2}$	$0.24\;\times \sigma_{Y}^{2}$	$0.18\;\times \sigma_{Y}^{2}$
$r_{XZ}=0.6$	$0.29\;\times \sigma_{Y}^{2}$	$0.23\;\times \sigma_{Y}^{2}$	$0.18\;\times \sigma_{Y}^{2}$	$0.13\;\times \sigma_{Y}^{2}$

From Table 3 we can see that, e.g., if the correlation between the response and explanatory variables is 0.8, the variance of the response variable in model G is 36% smaller than the original variance of the response variable; if the correlation is 0.7, about half of the variability is lost.

Table 4 gives numerical examples for the case of three modeling steps. Now, the decreased variance of the response variable is further accentuated. For example, when both correlations are 0.8, 59% of the original variance is lost; if both are 0.7, the corresponding figure is 76%.

Table 4 suggests that even if the correlation between explanatory variables and the response variable is fairly strong in the first two modeling steps, the last model is estimated using a response variable with substantially reduced variability compared to the response variable it is mimicking.

## Methods validation

We validated the correctness of the presented variance formulas for 3pHMB and 3pHHY prediction through Monte Carlo (MC) simulation. A real-world application of 3pHHY is presented by Varvia et al. [26].

An R Markdown file is available as supplementary material to this article. It provides an R code for the simulation with a step-by-step description of how the simulations were performed. The input information mimics boreal forest conditions in the northern part of Finland. The target variable is the AGBD ($Y_{i}$). The explanatory variable for model F is DBH ($X_{i})$; in model G it is an ALS variable related to vegetation height ($Z_{i}$), and in model Q, it is an ICESat-2 variable related to vegetation height ($P_{i}$). The superpopulation means ($\mu_{\left(\cdot \right)}$) and standard deviations ($sd_{\left(\cdot \right)})$ of the variables and the correlations between them are presented in Table 5. The sizes of the datasets are also given in the table.

**Table 5:** Input information.^2^

Description	Input values				
Superpopulation means	$\mu_{Y}$	$\mu_{X}$	$\mu_{Z}$	$\mu_{P}$	
	62.62	10.94	7.16	8.28	
Superpopulation standard deviations	$sd_{Y}$	$sd_{X}$	$sd_{Z}$	$sd_{P}$	
	49.53	6.30	4.34	5.69	
Superpopulation correlations between variables	$r_{YX}$	$r_{XZ}$	$r_{ZP}$		
	0.77	0.75	0.76		
Sizes of datasets involved in the analysis	$n_{I}$	$n_{II}$	$n_{III}$	$n_{IV}$	N
	102	943	1721	5760	38,400

^2^The input data mimics the conditions of the empirical case study presented in Varvia et al. [26]. Note that, although $n_{I}<n_{II}<n_{III}<n_{IV}<N$ in this case, the presented methodological framework does not require any specific dataset size relations.

The evaluation of the performance of the proposed predictors and variance formulas was conducted following the steps described below.

• *Step 1a:* Using the input information on the superpopulation means and standard deviations, the “true” model parameters and variances of variability terms in models F, G${}^{*}$, G, Q${}^{*}$ and Q were obtained following the theoretical outline presented in the section “Study of hierarchical modeling in the context of a superpopulation model”.
• *Step 1b:* The explanatory variables ($X_{I}$for model F over the dataset $n_{I}$, $Z_{II}$ for model G over the dataset $n_{II}$, $P_{III}$for model Q over the dataset $n_{III}$, and $P_{U}$ over U) were generated based on their mean values and standard deviations following normal distributions.
• *Step 1c:* The variances of the 3pHMB and 3pHHY predictors were computed according to Eqs. (8a) and (11) based on the results from steps 1a and 1b.

Steps 1a – 1c are preparations for the MC simulation, but located outside the MC loop, since they are repeated only once. The MC iterations included the following steps:

• *Step 2a:* The variability terms for the models F, G${}^{*}$, G, Q${}^{*}$ and Q were generated randomly based on Step 1a. As a consequence, the response variables for models were also generated for all elements in the datasets.
• *Step 2b:* The model parameters of the models F, G${}^{*}$, G, Q${}^{*}$ and Q were estimated based on the simulated data, and applied for predicting the AOI population mean following 3pHMB inference. The predicted value was recorded for each MC iteration.
• *Step 2c:* A simple random probability sample (without replacement) was drawn from $P_{U}$ and the AOI population mean was predicted following 3pHHY inference. Note that the predicted value was recorded for each MC iteration.
• *Step 2d:* Variance estimators were applied to estimate the variance of the 3pHMB and 3pHHY predictors based on the simulated outcomes from each MC iteration. The estimates were recorded for each MC iteration.

Steps 2a – 2d were repeated one million times. Based on the MC simulations, the empirical variances of the 3pHMB and 3pHHY predictors were obtained and could be compared with the variances according to the results from Eqs. (8a) and (11) as well as the corresponding variance estimators.

The results of the simulations are presented in Tables 6 and 7. In addition, it was observed that the average of the predicted AOI population means over the MC iterations based on the 3pHMB and 3pHHY predictors corresponded well with the AGBD superpopulation mean, which shows that the predictors are approximately unbiased.

**Table 6:** The variance of the 3pHMB and 3pHHY predictors and their corresponding estimated variances; $V({\hat{\bar{y}}}_{U(\cdot )})$ is the variance of the population mean predictor based on Eqs. (8a) and (11), $V_{MC}\left({\hat{\bar{y}}}_{U(\cdot )}\right)$ is the empirical variance of the population mean predictor from the MC iterations, and $\hat{V}\left({\hat{\bar{y}}}_{U(\cdot )}\right)$ is the average of estimated variances across the MC iterations.

3pHMB			3pHHY		
$V({\hat{\bar{y}}}_{U_{3pHMB}})$	$V_{MC}\left({\hat{\bar{y}}}_{U_{3pHMB}}\right)$	$\hat{V}\left({\hat{\bar{y}}}_{U_{3pHMB}}\right)$	$V({\hat{\bar{y}}}_{U_{3pHHY}})$	$V_{MC}\left({\hat{\bar{y}}}_{U_{3pHHY}}\right)$	$\hat{V}\left({\hat{\bar{y}}}_{U_{3pHHY}}\right)$
10.94	10.96	10.96	11.03	11.04	11.04

**Table 7:** The contribution of the terms involving traces to the overall variance of 3pHMB and 3pHHY predictors.

Predictor	Predictor variance based on Eq. (8a) and (11)	Terms involving traces	Relative contribution of terms involving traces
3pHMB	10.94	2.0 × 10^−3^	1.8 × 10^−2^%
3pHHY	11.03	0.8 × 10^−3^	0.7 × 10^−2^%

Table 6 shows the results from comparing the analytically derived variances of the predictors with the corresponding empirical variances from the MC iterations, and the average values of estimated variances. The Table shows that Eqs. (8a) and (11) are valid as variance formulas and that the corresponding variance estimators are approximately unbiased.

Referring to the Eqs. (8a) and (8b), it was suggested that terms with traces in (8a) could be neglected due to their small contribution to the overall variance of the 3pHMB and 3pHHY predictors. The empirical results in Table 7 validate this assertion.

## Conclusions

The main purpose of this study was to present predictors, variances, and variance estimators for hierarchical model-based inference and hierarchical hybrid inference involving three modeling steps. Previous studies have proposed solutions for methods involving two modeling steps (e.g., Saarela et al. [39]). The need for methods of this kind emanates from forest resources assessment utilizing multiple sources of remotely sensed data in combination with field data. In Monte Carlo simulations, the correctness of the proposed formulas was validated. In addition, it was shown that caution should be exercised when applying several modeling steps based on models with weak correlation between the explanatory variable(s) and the response variable, since multiple modeling steps dramatically reduce the variability of the predicted target variable, which may lead to problems in some applications. For example, a map produced on the basis of multiple modeling steps would display substantially reduced variability for the target variable compared to the real variability.

Although this article has focused on applications based on remotely sensed data, the methods are general and can be applied in any discipline where empirical data are lacking but proxies can be obtained through hierarchically nested models.

## CRediT authorship contribution statement

**Svetlana Saarela:** Conceptualization, Methodology, Software, Formal analysis, Writing – original draft, Writing – review & editing. **Petri Varvia:** Data curation, Methodology, Writing – review & editing. **Lauri Korhonen:** Data curation, Resources, Funding acquisition, Writing – review & editing. **Zhiqiang Yang:** Software, Writing – review & editing. **Paul L. Patterson:** Methodology, Writing – review & editing. **Terje Gobakken:** Resources, Writing – review & editing. **Erik Næsset:** Resources, Methodology, Writing – review & editing. **Sean P. Healey:** Resources, Funding acquisition, Writing – review & editing. **Göran Ståhl:** Methodology, Writing – original draft, Writing – review & editing.

## Declaration of Competing Interest

The authors declare that they have no known competing financial interests or personal relationships that could have appeared to influence the work reported in this paper.

## Data Availability

The data were simulated inside the validation code provided by the authors. An R Markdown file is attached as supplementary material.

## Footnotes

2 The input data mimics the conditions of the empirical case study presented in Varvia et al. [26]. Note that, although $n_{I}<n_{II}<n_{III}<n_{IV}<N$ in this case, the presented methodological framework does not require any specific dataset size relations.

## Footnotes

1 In the case of heterogeneity and correlation among model error terms, the generalized least square estimator should be applied to estimate the model parameters. Varvia et al. [26] presents an example where heterogeneous variance was taken into account in the context of 3pHHY.
